# Transhemispheric optic pathway degeneration following unilateral post-geniculate lesions

**DOI:** 10.1093/braincomms/fcag023

**Published:** 2026-01-28

**Authors:** Hinke N Halbertsma, Shereif Haykal, Hanna E Willis, Holly Bridge, Nomdo M Jansonius, Frans W Cornelissen

**Affiliations:** Laboratory of Experimental Ophthalmology, Department of Ophthalmology, University of Groningen, University Medical Center Groningen, Groningen, GZ 9713, the Netherlands; Laboratory of Experimental Ophthalmology, Department of Ophthalmology, University of Groningen, University Medical Center Groningen, Groningen, GZ 9713, the Netherlands; Oxford Centre for Integrative Neuroimaging, FMRIB, Nuffield Department of Clinical Neuroscience, University of Oxford, Oxford OX3 9DU, UK; Oxford Centre for Integrative Neuroimaging, FMRIB, Nuffield Department of Clinical Neuroscience, University of Oxford, Oxford OX3 9DU, UK; Laboratory of Experimental Ophthalmology, Department of Ophthalmology, University of Groningen, University Medical Center Groningen, Groningen, GZ 9713, the Netherlands; Laboratory of Experimental Ophthalmology, Department of Ophthalmology, University of Groningen, University Medical Center Groningen, Groningen, GZ 9713, the Netherlands

**Keywords:** optic pathways, post-geniculate lesion, transhemispheric degeneration, fixel-based analysis, diffusion-weighted imaging

## Abstract

A unilateral lesion in a post-geniculate section of the retino-geniculo-striate pathway (hereafter: optic pathway) leads to binocular vision loss in the contralateral visual hemifield. The extent of additional damage following such a lesion is not fully understood. Although degeneration of the ipsilesional optic tract and both retinas has been reported, potential degeneration in the contralesional post-chiasmal optic pathway has largely been overlooked. We aimed to investigate the presence and extent of contralesional degeneration in individuals with post-geniculate optic pathway lesions.

In this case-control study, we examined the optic pathways of study cohorts with 10 (dataset 1) and 22 (dataset 2) individuals with unilateral post-geniculate lesions and 12 (dataset 1) and 17 (dataset 2) neurologically healthy controls. For both datasets, we applied a higher-order analysis framework, i.e. fixel-based analysis, to diffusion-weighted imaging data to evaluate the white matter of optic pathway tracts.

Fixel-based analysis showed reduced fibre density and fibre-bundle cross-section in the ipsi- and contralesional optic pathways. Post-hoc analysis and observations further demonstrated reduced fibre density and fibre-bundle cross-section in the forceps major.

In individuals with unilateral post-geniculate optic pathway lesions, degeneration extends beyond their primary site to the optic pathway tracts, including contralesional ones. This pattern of widespread transhemispheric degeneration suggests that it spreads more extensively than previously recognized and highlights the need for understanding its implications for visual function.

## Teaser

Halbertsma *et al*. report widespread and transhemispheric white matter degeneration following unilateral post-geniculate optic pathway lesions, evidenced by axonal loss and fibre bundle atrophy. Their findings challenge the assumption that the contralesional hemisphere remains unaffected and underscore the need to investigate functional and rehabilitative implications of this degeneration.

## Graphical Abstract

**Graphical Abstract:**
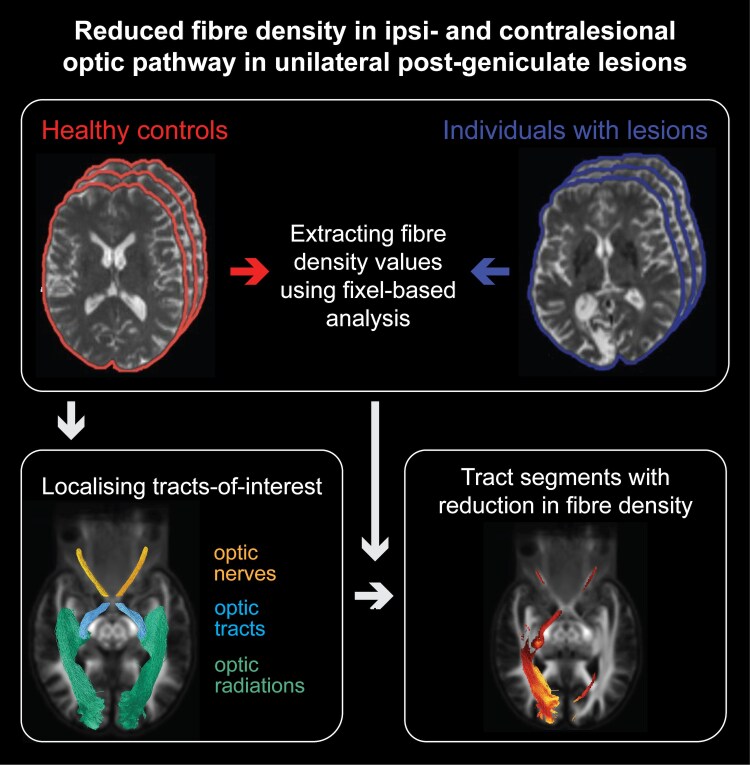

## Introduction

Unilateral damage to the post-geniculate retino-geniculo-striate pathway (hereafter: optic pathway), e.g. due to stroke or tumour (resection), causes vision loss in the contralateral visual hemifield or ‘(partial) hemianopia’. In individuals with such unilateral post-geniculate optic pathway lesions, further degeneration beyond the primary lesion site has been observed. This includes both degeneration of the ipsilesional optic tract^1-5^ and lateral geniculate nucleus^6^ and thinning of the retina,^2,7-20^ with the latter showing a topographic correspondence to the hemianopic visual field defect. Such additional damage is thought to be induced by transsynaptic degeneration, a process in which the degeneration spreads from damaged neurons to anatomically connected, yet previously undamaged, neurons. To date, the possibility that transsynaptic degeneration extends to the contralesional post-chiasmal optic pathway has generally been overlooked. Meanwhile, individuals with (partial) hemianopia may also experience perceptual deficits in their intact hemifield,^21-28^ suggesting that their contralesional post-chiasmal optic pathway may also be affected.

**Figure 1:**
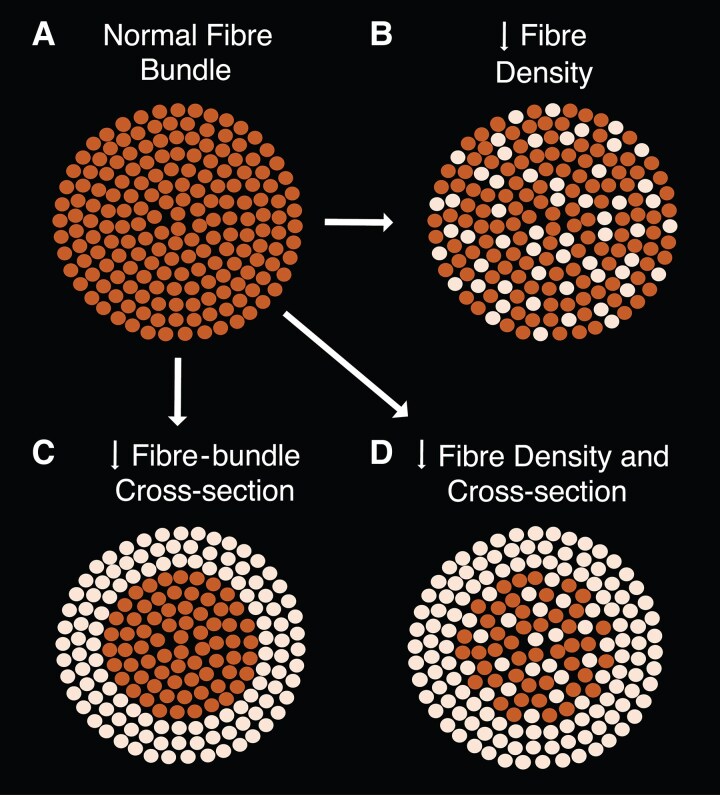
**Cross-section illustrations of white matter fibre bundles, depicting different patterns of neurodegeneration based on three quantitative fixel-based analysis metrics: fibre density, fibre-bundle cross-section, and their combination.** Each dot represents an individual axon. (**A**) Normal fibre bundle. (**B**) Fibre bundle with reduced fibre density, reflecting a loss of axons across the entire bundle. (**C**) Fibre bundle with reduced cross-section, reflecting fibre-bundle atrophy. (**D**) Fibre bundle with reduced fibre density and cross-section, reflecting both axonal loss and atrophy.

In this case-control study, we aimed to investigate the extent of transsynaptic degeneration in individuals with post-geniculate optic pathway lesions by comparing those with unilateral post-geniculate optic pathway lesions to healthy control individuals. We initially examined the entire bilateral optic pathway, from the eyes to the visual cortex, using a dataset with high-quality diffusion-weighted imaging (DWI) data. This allowed us to analyse the white matter properties of the bilateral optic pathway tracts—including the optic nerve, optic tract, and optic radiations—using a fixel-based analysis (FBA).^29^ FBA, a higher-order DWI analysis framework, enabled us to quantify distinct white matter fibre populations within the same voxel (‘fixels’) in terms of fibre density (Fig. 1B) and fibre-bundle cross-section (Fig. 1C).

We hypothesized finding degeneration beyond the primary lesion site, evident from reduced fibre density or fibre-bundle cross-section in the optic pathway tracts. Furthermore, we hypothesized an association between these patterns of degeneration and the time-since-injury^2-4,13,17^ and total lesion size.

Following the observations from this first dataset, we analysed a second independent DWI dataset, allowing us to re-examine the white matter in the post-chiasmal optic pathway, specifically the optic tract and optic radiations. This is the first study to use an FBA of DWI data to investigate the white matter integrity of the entire optic pathway in individuals with unilateral post-geniculate optic pathway lesions, and that includes replication of post-chiasmal findings using a second, independent dataset.

## Materials and methods

### Ethical approval

This study involved the analysis of two independent datasets. Ethical approval was obtained from the Medical Ethical Board of the University Medical Center Groningen (dataset 1) and University of Oxford Central Research Ethics Committee (R60132/RE001; R59810/RE001; dataset 2) and adhered to the tenets of the Declaration of Helsinki. All participants provided written informed consent before participation.

### Participants

Cohort 1 included 10 individuals (four females, mean age = 53.2 years) with a unilateral post-geniculate optic pathway lesion accompanied by a chronic (partial) hemianopia (hereafter referred to as hemianopic participants; two left-sided). Additionally, we included 12 neurologically healthy control participants (five females, mean age = 52.6 years) with intact visual fields. The two groups did not differ significantly in age (*t*(20) = 0.14, *P* = 0.89; independent *t*-test).

Participants in cohort 1 with any reported ophthalmic disorders (other than refractive errors), neurological disorders, or contraindications for MRI (e.g. vascular clips or claustrophobia) were excluded from the study. All participants underwent MRI scanning, retinal imaging and visual field examinations (see Supplementary Fig. 1)

Cohort 2 included 22 hemianopic participants (7 females, mean age = 51.5 years, ten left-sided) and 17 neurologically healthy control participants (7 females, mean age = 46.4 years) with reported intact visual fields. The two groups did not differ significantly in age (*t*(38) = 1.10, *P* = 0.28; independent *t*-test). Diffusion data for a subset of cohort 2 have already been published elsewhere and did not include a fixel-based analysis.^30^

Participants in cohort 2 with any diagnosed cognitive or psychiatric disorders, including executive or attentional deficits, or history of eye disease or impairment other than visual field deficits, including all forms of visuospatial neglect, were excluded from the study. All participants underwent MRI scanning.

The demographics and clinical characteristics of all hemianopic participants of cohorts 1 and 2 are provided in Table 1.

**Table 1:** Demographics and clinical characteristics of the hemianopic participants of cohort 1 (GRx) and cohort 2 (OXx)

	Sex	Age range (yrs)	Lesion aetiology	Lesioned hemisphere	Time-since-injury (months)	Total lesion size (mm^3^)
*Cohort 1*						
* GR01*	m	50–59	Stroke	right	13	-
* GR02*	m	60–69	Stroke	right	22	-
* GR03*	f	50–59	Stroke	right	161	-
* GR04*	m	40–49	Tumour resection	right	211	-
* GR05*	f	50–59	Stroke	right	341	-
* GR06*	m	60–69	Stroke	right	297	-
* GR07*	m	60–69	Stroke	right	25	-
* GR08*	m	40–49	Stroke	left	110	-
* GR09*	f	30–39	Tumour resection	right	26	-
* GR10*	f	40–49	Stroke	left	38	-
*Cohort 2*						
* OX01*	m	50–59	Stroke	right	68	7206
* OX02*	f	20–29	Stroke	left	39	27 009
* OX03*	f	50–59	Stroke	right	31	74 128
* OX04*	m	60–69	Stroke	right	21	1844
* OX05*	m	20–29	Stroke	right	13	51 706
* OX06*	m	40–49	Stroke	right	7	5013
* OX07*	m	40–49	Stroke	right	32	14 919
* OX08*	m	30–39	Stroke	right	25	2782
* OX09*	m	60–69	Stroke	left	47	5931
* OX10*	m	60–69	Stroke	right	7	5209
* OX11*	m	70–79	Stroke	right	58	60 775
* OX12*	m	70–79	Stroke	left	26	32 712
* OX13*	f	30–39	Stroke	left	30	20 766
* OX14*	f	30–39	Stroke	left	29	8935
* OX15*	m	60–69	Stroke	left	6	16 959
* OX16*	m	30–39	Stroke	left	26	16 371
* OX17*	f	40–49	Stroke	right	31	11 339
* OX18*	f	40–49	Stroke	right	297	1864
* OX19*	m	40–49	Stroke	left	14	23 736
* OX20*	m	60–69	Stroke	left	49	13 848
* OX21*	m	60–69	Stroke	right	13	8676
* OX22*	f	60–69	Stroke	left	37	90 611

### Ophthalmic data

Visual field examinations were conducted to confirm the presence of visual field defects in hemianopic participants in both cohorts, and to verify the absence of such defects in control participants. For cohort 1 only, structural imaging of the macula and optic nerve head was performed using OCT (Canon HS100 SD-OCT, Tokyo, Japan, see Supplementary Fig. 1) to assess structural integrity of the retina (see Supplementary Fig. 2).

### MRI data

#### Acquisition

For cohort 1, MRI data (dataset 1) were acquired at UMCG using a Siemens MAGNETOM Prisma 3T scanner with a 64-channel head coil. Two multi-shell diffusion-weighted imaging (DWI) scans (*b* = 1000 s/mm² and *b* = 2500 s/mm²) and three non-diffusion-weighted images (*b* = 0 s/mm²) were collected in two phase-encoding directions (anterior–posterior and posterior–anterior) with 2.0 mm isotropic voxels (TE = 85 ms, TR = 5500 ms, FOV = 210 × 210 × 132 mm, 66 slices, 64 gradient directions). In addition, a high-resolution whole-head T1-weighted MPRAGE scan was collected (voxel size = 1.0 mm isotropic, TE = 2.98 ms, TR = 2300 ms, FOV = 256 mm^3^, flip angle = 9 deg).

For cohort 2, MRI data (dataset 2) were acquired at the Oxford Centre for Integrative Neuroimaging, University of Oxford, using a Siemens Prisma 3T scanner with a 64-channel head coil. The UK Biobank sequence was used to acquire DWI data. A multi-shell (*b* = 1000 s/mm² and *b* = 2000s/mm²) image was collected in the anterior-posterior direction with 2.0 mm isotropic voxels (TE = 92 ms, TR = 3600 ms, FOV = 210 mm^3^; 72 slices, 104 gradient directions). Five *b*0 volumes were acquired without diffusion weighting (*b*-value = 0 s/mm^2^) and three b0 volumes with posterior-anterior encoding (*b*-value = 0 s/mm^2^) to correct for image distortion. In addition, a high-resolution whole-head T1-weighted MPRAGE scan was collected (voxel size = 1.0 mm isotropic, TE = 3.97 ms, TR = 1900ms, FOV = 192 mm^3^, flip angle = 8 deg).

#### Preprocessing

To investigate white matter degeneration of the post-retinal optic pathway at a group level, we flipped the MR volumes of the two (dataset 1) and ten (dataset 2) hemianopic participants with a left-sided optic pathway lesion along the mid-sagittal plane, such that the right hemisphere was the lesioned hemisphere for all hemianopic participants. To match this, we did the same for three (dataset 1) and seven (dataset 2) control participants.

DWI data were pre-processed using the software package MRtrix3 (version 3.0.2). The preprocessing steps included denoising, Gibbs ringing removal, motion and distortion correction (including eddy current distortions and susceptibility-induced EPI distortions), and bias field correction.

For the participants included in both dataset 1 and dataset 2, the estimated intracranial volume was computed by FreeSurfer from the T1-weighted images.^31^ Furthermore, for the hemianopic participants included in dataset 2, total lesion size was quantified using the T1-weighted images as part of a previous study (see Supplementary Methods—Lesion definition).^30^

#### Fixel-based analysis

We performed a fixel-based analysis (FBA) using MRtrix3 with the recommended FBA pipeline settings^32^ unless specified otherwise. FBA provides three structural measures of white matter: fibre density, fibre-bundle cross-section, and their combination (Fig. 1). In this study, we limited our analysis to fibre density and fibre-bundle cross-section.

For each participant, we: (i) up-sampled preprocessed DWI data to 1.25 mm isotropic voxels; (ii) created a brain mask and, for dataset 1 only, extended it to include the optic nerves; (iii) applied multi-tissue spherical deconvolution^33^ to generate a fibre orientation distribution (FOD) image; and (iv) performed bias field correction and intensity normalization on the FOD image. Using control participants’ (extended) brain masks and normalized FOD images, we generated unbiased, study-specific population FOD templates. These were segmented to estimate fibre-specific fixels. For each participant, we then: (i) produced nonlinear warps to register their FOD image to the template; (ii) segmented their FOD image to estimate fixels and apparent fibre density; (iii) assigned these fixels to the template fixels; and (iv) computed fibre-bundle cross-section across all fixels.

Finally, we conducted whole-brain probabilistic fibre tractography on the FOD templates, generating 20 million streamlines, filtered down to 2 million using spherical-deconvolution informed filtering.^34^ We used the resulting whole-brain tractograms to create fixel-to-fixel connectivity matrices for smoothing fibre density and fibre-bundle cross-section data.

#### Optic pathway fibre tracking

We used MRtrix’s probabilistic tractography algorithm to reconstruct the optic pathway tracts on the FOD population templates of dataset 1 and dataset 2.

**Figure 2:**
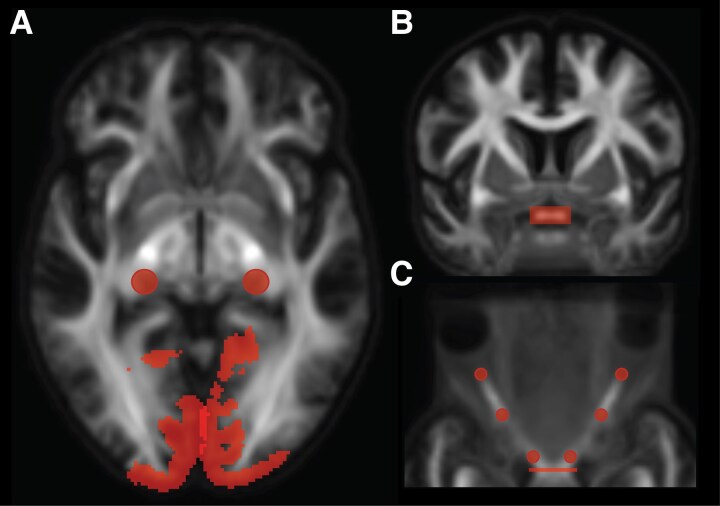
**Regions-of-interest placement for probabilistic tractography of the optic pathway tracts using the population template of dataset 1.** (**A**) Regions-of-interest (ROIs) used for optic radiation tracking, overlaid on a representative axial slice. Two seed ROIs (4-mm radius spheres) positioned at the lateral geniculate nuclei and two V1 target ROIs derived from FreeSurfer’s default Brodmann area parcellation. (**B)** and **(C**) ROIs used for anterior optic pathway tracking. An inclusion ROI positioned at the optic chiasm (rectangle measuring 6 × 14 × 1 voxels), overlaid on a representative coronal (**B**) and axial (**C**) slice, and three inclusion ROIs (2-mm radius spheres) placed along the length of both optic nerves overlaid on a representative axial (**C**) slice. For optic pathway tracking using the population template of dataset 2, the ROIs were placed similarly to those in panels **A** and **B**.

**Figure 3:**
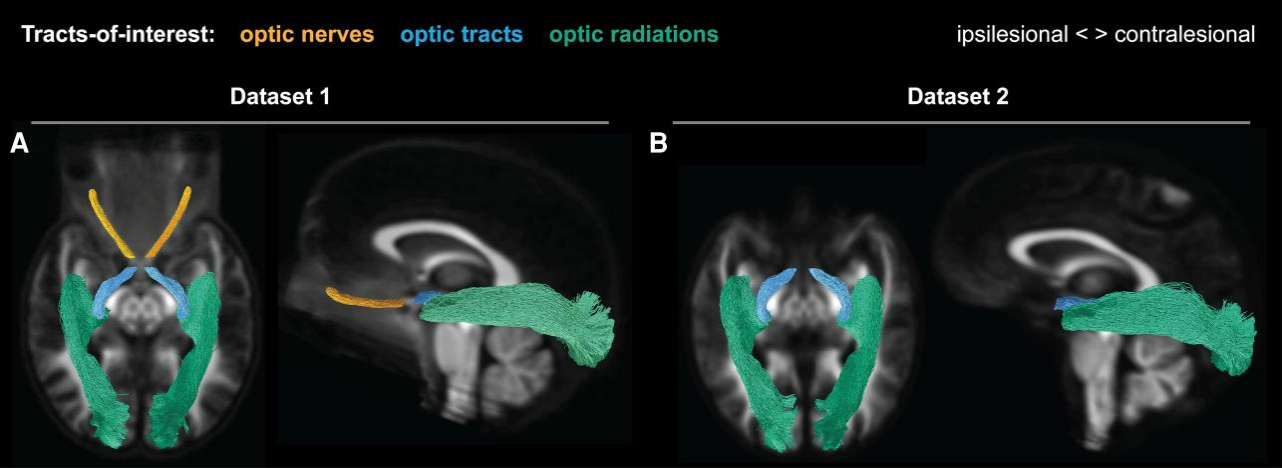
**Probabilistic tractography of optic pathways.** Streamlines resulting from the probabilistic tracking of the tracts-of-interest on the fibre orientation distribution population template of dataset 1 (**A**) and dataset 2 (**B**). Tracts-of-interest are the optic nerves (dataset 1 only), optic tracts, and optic radiations. All tracts-of-interest are shown in their full extent and overlaid on a representative axial slice of the population templates.

For dataset 1, we reconstructed the anterior optic pathways by tracking fibres between the eyes and the lateral geniculate nuclei. For this, we placed a 4-mm radius spherical seed regions-of-interests (ROIs) at the locations of both lateral geniculate nuclei (Fig. 2A; as described by Haykal *et al.*, 2019),^35^ a rectangular inclusion ROIs (measuring 6 × 14 × 1 voxels) at the optic chiasm (Fig. 2B), and three 2-mm radius spherical inclusion ROIs centrally along the length of both optic nerves (Fig. 2C; as described by Haykal *et al.*, 2020).^36^ We generated 500 streamlines for each lateral geniculate nucleus-eye combination, which we grouped to reconstruct the anterior optic pathway. The anterior optic pathway was then subdivided into the optic nerves (Fig. 3A, orange streamlines) and optic tracts (Fig. 3A, blue streamlines) for further analysis. For dataset 2, due to data acquisition constraints, we were unable to track the full anterior optic pathway. Specifically, multishell data was collected only in the anterior–posterior direction and only *b*0 volumes in the posterior-anterior direction. While the posterior–anterior *b*0 volumes allowed for some susceptibility distortion correction, this setup was less effective compared to dataset 1, where multishell data were collected in both anterior–posterior and posterior–anterior directions. Having full diffusion data in both directions provides more accurate susceptibility correction, especially in regions near air-tissue interfaces such as the orbitofrontal cortex, affecting the optic nerve fibres. However, the fibres between the optic chiasm and the lateral geniculate nuclei were less affected by these distortions and were successfully tracked to reconstruct the optic tracts (Fig. 3B, blue streamlines). Specifically, 1000 streamlines were generated using seed and inclusion ROIs at the optic chiasm and both lateral geniculate nuclei, consistent with the placement used in dataset 1.

For both datasets, we successfully reconstructed the optic radiations (Fig. 3A and B, green streamlines) by tracking fibres between the lateral geniculate nucleus and the primary visual cortex (V1) (Fig. 2A). We created V1 target ROIs by transforming a left and right V1 ROI in MNI-152 space, derived from FreeSurfer’s default Brodmann area parcellation, to the FOD template spaces. Using the lateral geniculate nucleus seed ROIs and the V1 target ROIs, we generated 10000 streamlines for both the left and right optic radiation. Fibre tracking was anatomically constrained using optic radiation masks derived from the Juelich histological atlas.^37^ The resulting optic pathway tracts were converted to fixel-masks, allowing fixel-wise tract-of-interest analyses.

#### Fixel-wise tract-of-interest statistical analyses

For each tract-of-interest (i.e. the optic nerves, optic tracts, and optic radiations), we performed a fixel-wise comparison of participants’ smoothed fibre density and fibre-bundle cross-section data. Statistical analyses were performed on the participants’ fixels within the tract-of-interest fixel masks. Differences between the hemianopic and control participants were tested for significance using a general linear model, with demeaned sex and age as covariates. To account for potential confounding effects of intracranial volume on the fibre-bundle cross-section measures,^29,38^ we included *z*-scored, sex-adjusted, intracranial volume residuals (see Supplementary Methods—Intracranial volume correction) as an additional covariate. This adjustment was applied to both datasets, given previously reported sex differences in intracranial volume^39^ even though the correlation between intracranial and fibre-bundle cross-section reached significance only in dataset 2 (*r* = 0.36, *P* = 0.02) and not in dataset 1 (*r* = 0.33, *P* = 0.13). For each fixel within the tract-of-interest fixel mask, we obtained a familywise error-corrected *P*-value via nonparametric permutation testing (*n* = 5000).

Furthermore, for each tract-of-interest beyond the lesion site (i.e. optic nerves, optic tracts, and contralesional optic radiation) that showed a significant difference between hemianopic and control participants, we calculated the mean reduction in fibre density and fibre-bundle cross-section across the affected fixels for each hemianopic participant. We conducted Pearson linear partial correlation to evaluate the relationship between these mean reductions and (i) the time since injury (in months, log-transformed) for both datasets, and (ii) the total lesion size for dataset 2 only. Age (in years) was included as a covariate, given the known age-related degeneration of WM.^40^

## Results

### Reduced fibre density and fibre-bundle cross-section in the bilateral optic pathway tracts

**Figure 4:**
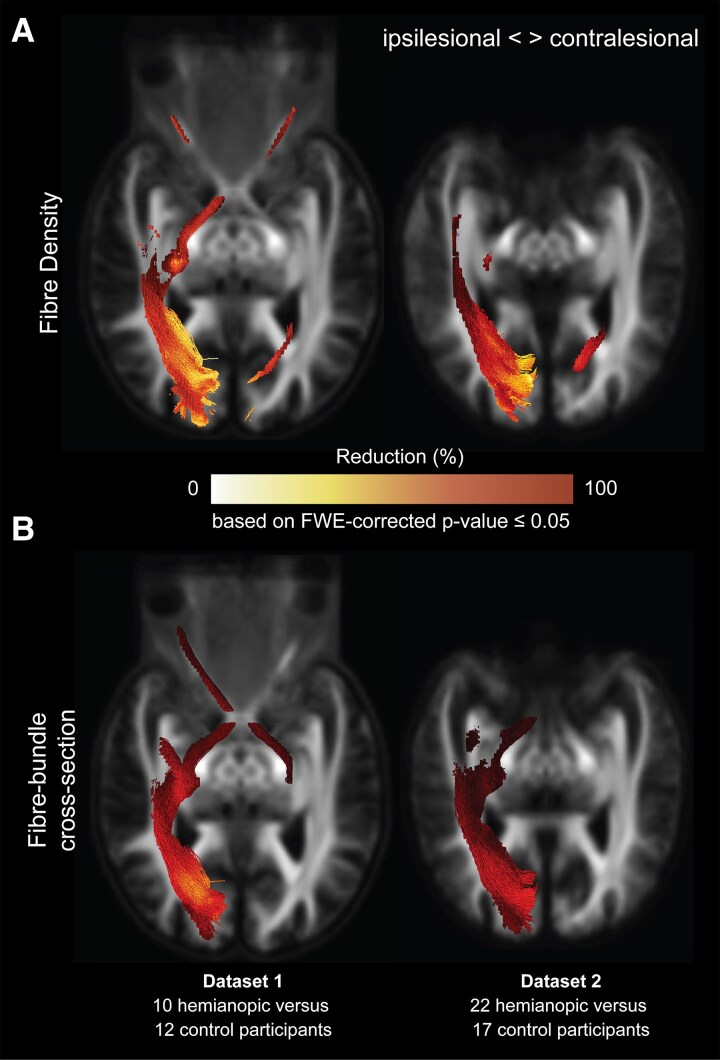
**Reductions in fibre density and fibre-bundle cross-section in hemianopic individuals in the ipsilesional and contralesional tracts-of-interest.** Statistical significance was assessed using nonparametric permutation testing (*n* = 5000), with family-wise error (FWE) correction applied across fixel-wise comparisons. The figure shows only the streamline segments of the tracts-of-interest corresponding to fixels with statistically significant reductions (family-wise error-corrected *P* ≤ 0.05) in hemianopic participants for fibre density (**A**) and fibre-bundle cross-section (**B**). Reductions are presented as percentages relative to control participants for dataset 1 (top row; 10 hemianopic participants versus 12 controls) and dataset 2 (bottom row; 22 hemianopic participants versus 17 controls). All streamline segments are shown in their full extent and overlaid on a representative axial slice of the population templates. Ipsilesional data are presented in the left hemisphere.

For both datasets, we observed significant reductions in fibre density and fibre-bundle cross-section for the hemianopic compared to the control participants, in both the ipsi- and contralesional tracts (Fig. 4).

In dataset 1, as expected given the primary lesion site, significant reductions were observed in a large portion of the ipsilesional optic radiation, with reductions up to 94% in fibre density and up to 73% in fibre-bundle cross-section. Reductions extended beyond the primary lesion site. In fibre density, notable reductions were observed in the ipsilesional optic nerve (up to 40%), ipsilesional optic tract (up to 82%), contralesional optic nerve (up to 35%), and contralesional optic radiation (up to 82%). Reductions in fibre-bundle cross-section were observed in the ipsilesional optic tract (up to 36%), contralesional optic tract (up to 11%) and ipsilesional optic nerve (up to 12%).

In dataset 2, reductions were again prominent in the ipsilesional optic radiation, consistent with the primary lesion site, with reductions in fibre density of up to 87% and in fibre-bundle cross-section of up 39%. Beyond the primary lesion site, we observed reductions in fibre density in the contralesional optic radiation (up to 46%) and fibre-bundle cross-section in the ipsilesional optic tract (up to 12%).

Figure 4 illustrates these reductions by showing only the streamline segments of the tracts-of-interest where significant reductions were observed in fibre density (panel A) and fibre-bundle cross-section (panel B). Moreover, reductions in fibre density within the contralesional optic radiation in dataset 2 closely mirrored those observed in dataset 1, supporting the reproducibility of this finding across independent cohorts.

### Exploratory correlations between degeneration beyond the lesion site and clinical variables

**Figure 5:**
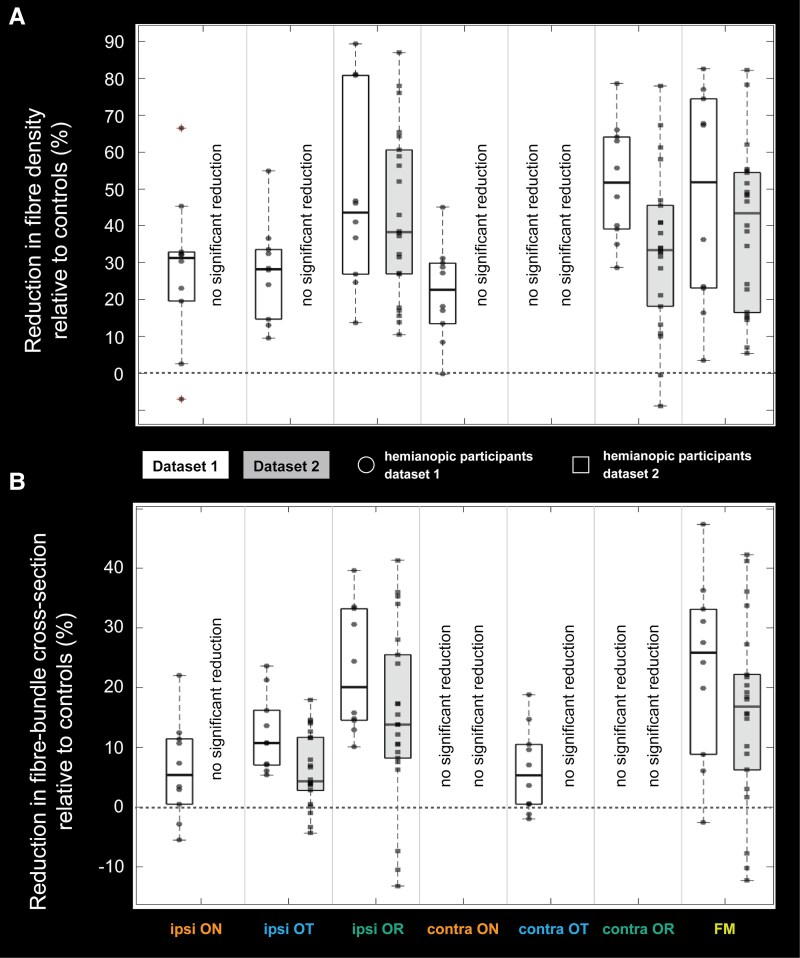
**Distribution of mean reductions in fibre density and fibre-bundle cross-section across the affected fixel cluster of each tract-of-interest.** Boxplots show the distribution of mean reductions in fibre density (**A**) and fibre-bundle cross-section (**B**) across hemianopic participants, relative to controls, for the cluster of significantly affected fixels within each tract of interest (optic nerve (ON), optic tract (OT) and optic radiations (OR), both ipsilesional (ipsi) and contralesional (contra), and the forceps major (FM)). Black dots (dataset 1, *n* = 10) and squares (dataset 2, *n* = 22) indicate the mean reduction for each hemianopic participant. The dashed line at 0 indicates no difference from controls; negative values indicate an increase relative to controls. Tracts without significant reductions across hemianopic participants, relative to controls (see also Fig. 4), are labelled ‘no significant reduction’.

In either dataset, no significant correlations were found between the hemianopic participants’ time-since-injury and mean reductions in fibre density or fibre-bundle cross-section for any tract. In dataset 2, total lesion size was significantly correlated with reductions in fibre-bundle cross-section only in the ipsilesional optic radiation (r = 0.54, *P* = 0.01). Figure 5 shows boxplots for each tract-of-interest, representing the distribution of hemianopic participants’ mean reductions, across the cluster of affected fixels, in fibre density (panel A) and fibre-bundle cross-section (panel B) relative to the control participants.

### Post-hoc analyses

#### Reduced fibre density and fibre-bundle cross-section along the forceps major

**Figure 6:**
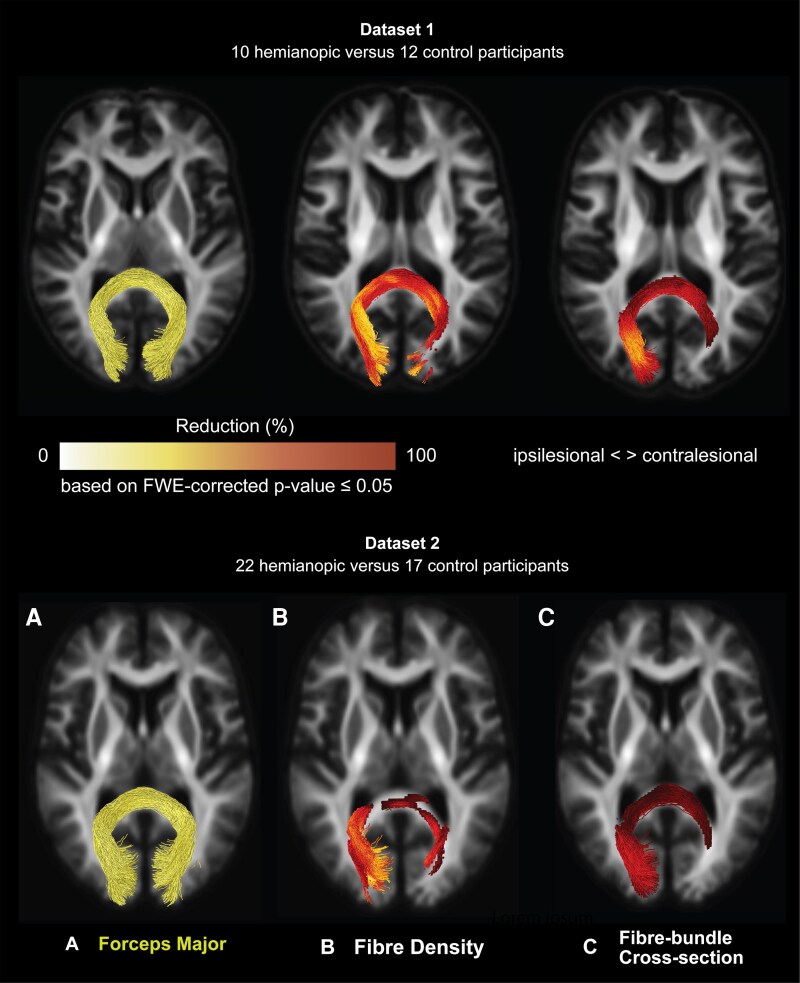
**Probabilistic tractography of the forceps major and associated reductions in fibre density and fibre-bundle cross-section. (A)** Streamlines resulting from the probabilistic tracking of the forceps major for dataset 1 (top) and dataset 2 (bottom). (**B, C)**. Statistical significance was assessed using nonparametric permutation testing (*n* = 5000), with family-wise error (FWE) correction applied across fixel-wise comparisons. The figure shows only the streamline segments of the tracts-of-interest corresponding to fixels with statistically significant reductions (family-wise error-corrected *P* ≤ 0.05) in hemianopic participants relative to controls for fibre density (**B**) and fibre-bundle cross-section (**C**). All streamline segments are shown in their full extent and overlaid on a representative axial slice of the population template. Ipsilesional data are presented in the left hemisphere.

To further explore the spatial specificity of these reductions, a post-hoc analysis was performed on the forceps major, a large white matter tract connecting bilateral V1 regions. To reconstruct the forceps major for both datasets, 1000 streamlines were generated bidirectionally between the V1 of both hemispheres (Fig. 6A), and fixel-wise comparisons were conducted as described in the methods. In datasets 1 and 2, the forceps major showed reductions in fibre density of up to 96% and 87%, respectively, and in fibre-bundle cross-section of up to 73% and 38%, respectively, when comparing hemianopic to control participants.

**Figure 7:**
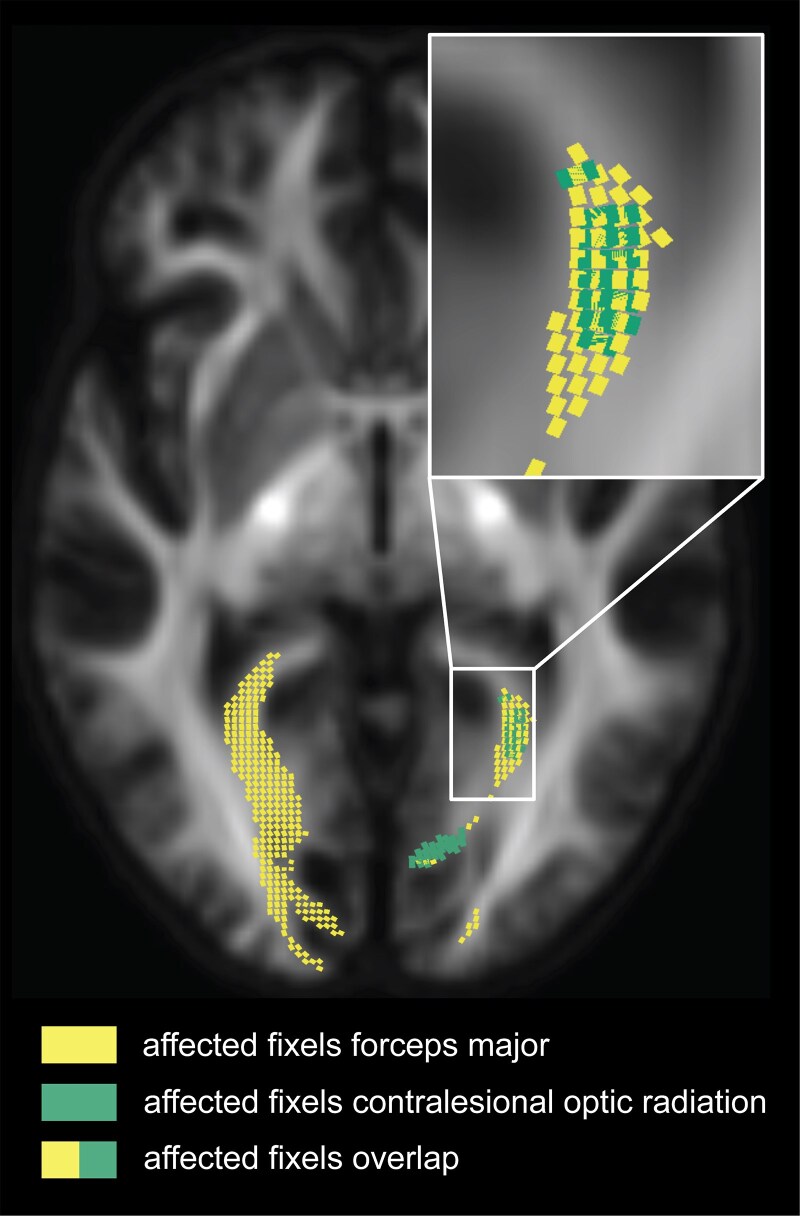
**Overlap between affected fixels in the forceps major and contralesional optic radiation.** Affected fixels in the forceps major (yellow) and the contralesional optic radiation (green) show areas of spatial overlap. Fixels cropped to a single axial slice (*x* = 54) are displayed. The zoomed-in section highlights the overlapping fixels.

Reductions in the forceps major were largest at the ipsilesional end of the tract, as expected given the lesion site, and extended into the contralesional hemisphere. Figure 6 B and C illustrate these reductions by showing only the streamline segments of the forceps major with significant reductions in fibre density (panel B) and fibre-bundle cross-section (panel C). A comparison of the affected fixels for the contralesional optic radiation and the forceps major reveals that fibre density reductions are found for primarily overlapping fixels (Fig. 7).

Additionally, we conducted a whole-brain analysis where we compared fixel values across the entire brain between the individuals with post-geniculate lesions and controls. No additional significant differences in fibre density or fibre cross-section were identified beyond our tracts of interest.

#### No relationship between reductions in the contralesional hemispheres and a set of clinically relevant measures

To explore whether the white matter degeneration in the contralesional hemisphere was associated with clinically relevant measures, additional post-hoc analyses were performed. Specifically, Pearson’s linear partial correlation analyses were conducted to examine the relationship between the mean reduction in fibre density in the contralesional optic radiation and two clinical measures: (i) a structural measure, namely thickness of the ganglion cell–inner plexiform layer in the unaffected hemifield (available for hemianopic participants in dataset 1; see Supplementary Results—Macular thinning and Supplementary Fig. 3), and (ii) a functional measure, the average total deviation in visual field sensitivity of the unaffected hemifield, derived from Humphrey Visual Field testing (data combined from datasets 1 and 2, see Supplementary Methods—Visual Field Sensitivity (Total Deviation)). Age (in years) was included as a covariate to account for known age-related white matter changes.^40^ Similar partial correlation analyses were conducted for reductions in fibre-bundle cross-section in dataset 1, using the same clinical measures. No significant correlations were found between the fixel-based reductions and either of the clinical variables tested.

## Discussion

We found widespread degeneration in the optic pathway of individuals with unilateral post-geniculate lesions that extends beyond the primary lesion site. Notably, this is the first study to demonstrate degeneration not only in the ipsilesional optic pathway but also in the contralesional optic pathway and the forceps major. Replicating this transhemispheric degeneration across two independent cohorts underscores its robustness and reproducibility. No relationships were found between the extent of degeneration and various clinically relevant measures. Below, we discuss these findings in more detail.

### Transsynaptic degeneration along optic pathways

Using a fixel-based analysis (FBA) across two independent cohorts, we identified significant reductions in fibre density and fibre-bundle cross-section along both the ipsilesional and contralesional optic pathway. These metrics reflect changes in distinct tissue properties: reduced fibre density is commonly interpreted as axonal loss, while reduced fibre-bundle cross-section reflects tract atrophy.

As expected, the largest reductions in both FBA metrics were consistently observed in the ipsilesional optic radiation, aligning with unilateral post-geniculate damage. Degeneration in the ipsilesional optic tract, bilateral optic nerves and retinas (see Supplementary Results and Supplementary Figs 3 and 4) supports a pattern of retrograde transsynaptic degeneration spreading from the lesion site towards the eyes.^1-16,41^ Additionally, the degeneration in the forceps major, a white matter tract anatomically connecting the occipital lobes, suggests propagation along visual white matter into the contralesional hemisphere.

Previous neuroimaging studies focused primarily on ipsilesional degeneration, often reporting ipsi-/contralesional asymmetries in white matter volume^1-5^ or fractional anisotropy.^6^ By relying on within-subject asymmetries, these approaches typically omitted contralesional effects. Our study compared hemianopic participants to age-matched controls and also revealed degeneration in the contralesional optic tract and optic radiation. Importantly, fibre density reductions in the contralesional optic radiation were spatially consistent across both cohorts, reinforcing the transhemispheric impact of the unilateral post-geniculate lesion.

Furthermore, our use of the higher-order analysis framework FBA is an analytical improvement for detecting and interpreting white matter degeneration compared to previous work employing conventional DWI frameworks, such as diffusion tensor imaging. While diffusion tensor imaging assumes a single diffusion direction per voxel, FBA models multiple fibre populations via fibre orientation distributions.^42^ This enables accurate analysis of complex fibre architecture and offers biologically meaningful metrics that distinguish microstructural (e.g. axonal loss) from macrostructural (e.g. fibre-bundle atrophy) changes, offering a more nuanced understanding of white matter degeneration.

We found no significant correlations between degeneration and total lesion size or time since injury, except for the expected association between total lesion size and degeneration in the ipsilesional optic radiation. This suggests that these clinical variables may not reliably predict degeneration beyond the primary lesion site. Future longitudinal studies may better capture the dynamics of degeneration beyond the primary lesion site.

### Future directions and clinical implications

Although contralesional degeneration was consistently observed, its clinical relevance remains unclear. Post-hoc analyses revealed no significant associations with retinal thinning and visual field sensitivity. These exploratory results represent an initial step towards understanding the clinical implications of contralesional degeneration, which future studies should examine more directly. Clarifying the impact of contralesional degeneration is important, given previous on perceptual deficits in the perimetrically intact hemifield of individuals with unilateral post-geniculate lesions.^21-28^ The observed degeneration in the forceps major suggests the possibility of disrupted interhemispheric visual communication.^43^

Our findings may also inform rehabilitation. The extent of optic pathway degeneration may help explain inter-individual differences in rehabilitation potential. A prior study has shown that macrostructural atrophy (i.e. optic tract shrinkage)^3^ can predict limits to training-induced visual recovery in individuals with unilateral post-geniculate lesions. In the context of fixel-based analysis, these metrics map closely onto fibre-bundle cross-section and fibre density, respectively. Further research should assess whether these FBA metrics can serve as biomarkers for rehabilitation potential.

Additionally, previous findings that retinal thinning stabilizes with visual training in the blind field^7^ suggest possible neuroprotective effects of behavioural interventions. Based on this, we speculate that rehabilitation targeting both the blind and intact hemifield may help preserve retinal integrity and mitigate the spread of degeneration.^44^

### Limitations

Despite the strengths of this study, including a relatively large sample size of 30 + hemianopic participants, high-quality diffusion data, and replication across independent cohorts, several limitations should be acknowledged.

First, there was a spatial overlap between affected fixels in the contralesional optic radiation and forceps major, making it difficult to attribute degeneration to one tract specifically. Nonetheless, the consistent presence of contralesional degeneration supports a transhemispheric impact of unilateral post-geniculate lesions.

Second, we cannot fully exclude the potential influence of comorbidities in hemianopic participants. However, several factors argue against this explanation. For transsynaptic degeneration to be observed at the group level, comorbidities would need to produce consistent patterns of degeneration, which is unlikely given the heterogeneity of the hemianopic participants. Moreover, no participants had documented neurodegenerative disorders or bilateral optic pathway lesions. Minor ipsilesional visual field defects observed in two hemianopic participants (GR07 and GR10, Supplementary Fig. 1) could suggest comorbidities affecting the contralesional pathway. However, these individuals did not exhibit the largest reductions in fibre density in the contralesional optic radiation (35% and 66%, respectively; cohort range: 29–78%) nor the largest reductions in fibre-bundle cross-section values in the contralesional optic tract (−1% and 10%, respectively, cohort range: −2–19%). These minor ipsilesional visual field defects may also reflect normal variability and reliability issues in subjective visual field assessments, such as Humphreys’ field analysis. Nevertheless, future studies should incorporate additional screening to identify potential comorbidities more definitively.

Third, although lesions were classified as post-geniculate based on MR image inspection, we cannot entirely rule out damage to the ipsilesional lateral geniculate nucleus. However, such damage alone would not explain the transhemispheric patterns of degeneration observed.

## Conclusions

We are the first to demonstrate widespread and transhemispheric white matter degeneration following unilateral post-geniculate optic pathway lesions, evidenced by axonal loss and fibre bundle atrophy. The finding of transhemispheric degeneration highlights that the contralesional hemisphere should not be assumed to be unaffected and supports normal vision. Future studies should explore the functional implications of this widespread degeneration as well as its implications for rehabilitation.

## Supplementary Material

fcag023_Supplementary_Data

## Data Availability

The data and code that support the findings of this study are available from the corresponding author, upon reasonable request.

## References

[1] Bridge H, Jindahra P, Barbur J, Plant GT. Imaging reveals optic tract degeneration in hemianopia. Invest Ophthalmol Vis Sci. 2011;52(1):382–388. 20739474 10.1167/iovs.10-5708

[2] Cowey A, Alexander I, Stoerig P. Transneuronal retrograde degeneration of retinal ganglion cells and optic tract in hemianopic monkeys and humans. Brain. 2011;134(Pt 7):2149–2157. 21705429 10.1093/brain/awr125

[3] Fahrenthold BK, Cavanaugh MR, Jang S, et al Optic tract shrinkage limits visual restoration after occipital stroke. Stroke. 2021;52(11):3642–3650. 34266305 10.1161/STROKEAHA.121.034738 PMC8545836

[4] Millington RS, Yasuda CL, Jindahra P, et al Quantifying the pattern of optic tract degeneration in human hemianopia. J Neurol Neurosurg Psychiatry. 2014;85(4):379–386. 24163431 10.1136/jnnp-2013-306577

[5] Patel KR, Ramsey LE, Metcalf NV, Shulman GL, Corbetta M. Early diffusion evidence of retrograde transsynaptic degeneration in the human visual system. Neurology. 2016;87(2):198–205. 27306632 10.1212/WNL.0000000000002841 PMC4940065

[6] Kim Y, Im S, Oh J, Jung Y, Jun SY. Detection of post-stroke visual field loss by quantification of the retrogeniculate visual pathway. J Neurol Sci. 2022;439:120297. 35640329 10.1016/j.jns.2022.120297

[7] Fahrenthold BK, Cavanaugh MR, Tamhankar M, et al Training in cortically blinded fields appears to confer patient-specific benefit against retinal thinning. Invest Ophthalmol Vis Sci. 2024;65(4):29. 10.1167/iovs.65.4.29 PMC11033601 38635245

[8] Anjos R, Vieira L, Costa L, et al Macular ganglion cell layer and peripapillary retinal nerve fibre layer thickness in patients with unilateral posterior cerebral artery ischaemic lesion: An optical coherence tomography study. Neuroophthalmology. 2016;40(1):8–15. 27928376 10.3109/01658107.2015.1122814 PMC5123159

[9] Goto K, Miki A, Yamashita T, et al Sectoral analysis of the retinal nerve fiber layer thinning and its association with visual field loss in homonymous hemianopia caused by post-geniculate lesions using spectral-domain optical coherence tomography. Arbeitsphysiologie. 2016;254(4):745–756. 10.1007/s00417-015-3181-1 PMC4799802 26446718

[10] Herro AM, Lam BL. Retrograde degeneration of retinal ganglion cells in homonymous hemianopsia. Clin Ophthalmol. 2015;9:1057–1064. 26089638 10.2147/OPTH.S81749 PMC4468984

[11] Jaumandreu L, Sánchez-Gutiérrez V, Muñoz-Negrete FJ, de Juan V, Rebolleda G. Reduced peripapillary and macular vessel density in unilateral postgeniculate lesions with retrograde transsynaptic degeneration. J Neuroophthalmol. 2019;39(4):462–469. 31658224 10.1097/WNO.0000000000000794

[12] Jindahra P, Petrie A, Plant GT. Retrograde trans-synaptic retinal ganglion cell loss identified by optical coherence tomography. Brain. 2009;132(Pt 3):628–634. 19224900 10.1093/brain/awp001

[13] Jindahra P, Petrie A, Plant GT. The time course of retrograde trans-synaptic degeneration following occipital lobe damage in humans. Brain. 2012;135(Pt 2):534–541. 22300877 10.1093/brain/awr324

[14] Keller J, Sánchez-Dalmau BF, Villoslada P. Lesions in the posterior optic pathway promote trans-synaptic degeneration of retinal ganglion cells. PLoS One. 2014;9(5):e97444. 24857938 10.1371/journal.pone.0097444 PMC4032251

[15] Mitchell JR, Oliveira C, Tsiouris AJ, Dinkin MJ. Corresponding ganglion cell atrophy in patients with postgeniculate homonymous visual field loss. J Neuroophthalmol. 2015;35(4):353–359. 26035806 10.1097/WNO.0000000000000268

[16] Mühlemann F . Homonymous hemiatrophy of ganglion cell layer from retrochiasmal lesions in the optic pathway. Neurology. 2020;94:e323–e329. 31848256 10.1212/WNL.0000000000008738

[17] Yamashita T, Miki A, Goto K, et al Retinal ganglion cell atrophy in homonymous hemianopia due to acquired occipital lesions observed using Cirrus high-definition-OCT. J Ophthalmol. 2016;2016:2394957. 27274865 10.1155/2016/2394957 PMC4870342

[18] Shin HY, Park HYL, Choi JA, Park CK. Macular ganglion cell-inner plexiform layer thinning in patients with visual field defect that respects the vertical meridian. Arbeitsphysiologie. 2014;252(9):1501–1507. 10.1007/s00417-014-2706-3 25104464

[19] Yamashita T, Miki A, Goto K, et al Preferential atrophy of the central retinal ganglion cells in homonymous hemianopia due to acquired retrogeniculate lesions demonstrated using swept-source optical coherence tomography. Acta Ophthalmol. 2018;96(4):e538–e539. 29193786 10.1111/aos.13644 PMC6099321

[20] Lee JI, Boerker L, Gemerzki L, et al Retinal changes after posterior cerebral artery infarctions display different patterns of the nasal und temporal sector in a case series. Front Neurol. 2020;11:508. 32582017 10.3389/fneur.2020.00508 PMC7290045

[21] Bola M, Gall C, Sabel BA. Sightblind”: Perceptual deficits in the “intact” visual field. Front Neurol. 2013;4:80. 23805126 10.3389/fneur.2013.00080 PMC3691518

[22] Bola M, Gall C, Sabel BA. The second face of blindness: Processing speed deficits in the intact visual field after pre- and post-chiasmatic lesions. PLoS One. 2013;8(5):e63700. 23667657 10.1371/journal.pone.0063700 PMC3648511

[23] Cavézian C . Hemisphere-dependent ipsilesional deficits in hemianopia: Sightblindness in the “intact” visual field. Cortex. 2015;69:166–174. 26073147 10.1016/j.cortex.2015.05.010

[24] Poggel DA, Treutwein B, Strasburger H. Time will tell: Deficits of temporal information processing in patients with visual field loss. Brain Res. 2011;1368:196–207. 20974114 10.1016/j.brainres.2010.10.065

[25] Schadow J, Dettler N, Paramei GV, et al Impairments of Gestalt perception in the intact hemifield of hemianopic patients are reflected in gamma-band EEG activity. Neuropsychologia. 2009;47(2):556–568. 18996403 10.1016/j.neuropsychologia.2008.10.012

[26] Woutersen K, Geuzebroek AC, van den Berg AV, Goossens J. Useful field of view performance in the intact visual field of hemianopia patients. Invest Ophthalmol Vis Sci. 2020;61(5):43. 10.1167/iovs.61.5.43 PMC7405799 32446248

[27] Chokron S, Peyrin C, Perez C. Ipsilesional deficit of selective attention in left homonymous hemianopia and left unilateral spatial neglect. Neuropsychologia. 2019;128:305–314. 29551364 10.1016/j.neuropsychologia.2018.03.013

[28] Geuzebroek AC, van den Berg AV. Impaired visual competition in patients with homonymous visual field defects. Neuropsychologia. 2017;97:152–162. 28209521 10.1016/j.neuropsychologia.2017.02.011

[29] Raffelt DA, Tournier JD, Smith RE, et al Investigating white matter fibre density and morphology using fixel-based analysis. Neuroimage. 2017;144(Pt A):58–73. 27639350 10.1016/j.neuroimage.2016.09.029 PMC5182031

[30] Willis HE, Caron B, Cavanaugh MR, et al Rehabilitating homonymous visual field deficits: White matter markers of recovery-stage 2 registered report. Brain Commun. 2024;6(5):fcae323. 39429244 10.1093/braincomms/fcae323 PMC11487913

[31] Dale AM, Fischl B, Sereno MI. Cortical surface-based analysis. I. Segmentation and surface reconstruction. Neuroimage. 1999;9(2):179–194. 9931268 10.1006/nimg.1998.0395

[32] Tournier JD, Smith R, Raffelt D, et al MRtrix3: A fast, flexible and open software framework for medical image processing and visualisation. Neuroimage. 2019;202:116137. 31473352 10.1016/j.neuroimage.2019.116137

[33] Jeurissen B, Tournier JD, Dhollander T, Connelly A, Sijbers J. Multi-tissue constrained spherical deconvolution for improved analysis of multi-shell diffusion MRI data. Neuroimage. 2014;103:411–426. 25109526 10.1016/j.neuroimage.2014.07.061

[34] Smith RE, Tournier JD, Calamante F, Connelly A. SIFT: Spherical-deconvolution informed filtering of tractograms. Neuroimage. 2013;67:298–312. 23238430 10.1016/j.neuroimage.2012.11.049

[35] Haykal S, Curcic-Blake B, Jansonius NM, Cornelissen FW. Fixel-based analysis of optic pathway white matter in primary open-angle glaucoma. Invest Ophthalmol Vis Sci. 2019;60:3803–3812. 31504081 10.1167/iovs.19-27447

[36] Haykal S, Jansonius NM, Cornelissen FW. Investigating changes in axonal density and morphology of glaucomatous optic nerves using fixel-based analysis. Eur J Radiol. 2020;133:109356. 33129102 10.1016/j.ejrad.2020.109356

[37] Bürgel U, Amunts K, Hoemke L, Mohlberg H, Gilsbach JM, Zilles K. White matter fiber tracts of the human brain: Three-dimensional mapping at microscopic resolution, topography and intersubject variability. Neuroimage. 2006;29(4):1092–1105. 16236527 10.1016/j.neuroimage.2005.08.040

[38] Smith RE, Dhollander T, Connelly A. On the regression of intracranial volume in fixel-based analysis. In: *Conference: 27th International Society of Magnetic Resonance in Medicine*. 2019:3385. https://cds.ismrm.org/protected/19MProceedings/PDFfiles/3385.html

[39] Ritchie SJ, Cox SR, Shen X, et al Sex differences in the adult human brain: Evidence from 5216 UK biobank participants. Cereb Cortex. 2018;28(8):2959–2975. 29771288 10.1093/cercor/bhy109 PMC6041980

[40] Yap QJ, Teh I, Fusar-Poli P, Sum MY, Kuswanto C, Sim K. Tracking cerebral white matter changes across the lifespan: Insights from diffusion tensor imaging studies. J Neural Transm (Vienna). 2013;120(9):1369–1395. 23328950 10.1007/s00702-013-0971-7

[41] Sharma S, Chitranshi N, Wall RV, et al Trans-synaptic degeneration in the visual pathway: Neural connectivity, pathophysiology, and clinical implications in neurodegenerative disorders. Surv Ophthalmol. 2022;67(2):411–426. 34146577 10.1016/j.survophthal.2021.06.001

[42] Jeurissen B, Leemans A, Tournier JD, Jones DK, Sijbers J. Investigating the prevalence of complex fiber configurations in white matter tissue with diffusion magnetic resonance imaging. Hum Brain Mapp. 2013;34(11):2747–2766. 22611035 10.1002/hbm.22099 PMC6870534

[43] Berlucchi G . Visual interhemispheric communication and callosal connections of the occipital lobes. Cortex. 2014;56:1–13. 23489777 10.1016/j.cortex.2013.02.001

[44] Cavanaugh MR, Blanchard LM, McDermott M, Lam BL, Tamhankar M, Feldon SE. Efficacy of visual retraining in the hemianopic field after stroke: Results of a randomized clinical trial. Ophthalmology. 2021;128(7):1091–1101. 33242498 10.1016/j.ophtha.2020.11.020

