# Supplementary material for: Transhemispheric optic pathway degeneration following unilateral post-geniculate lesions
Source: Brain Commun. 2026 Jan 28;8(1):fcag023. doi: 10.1093/braincomms/fcag023 (PMC12926223; doi:10.1093/braincomms/fcag023)
Supplement: fcag023_Supplementary_Data [file fcag023_supplementary_data.docx]

# Supplementary Material

# Analysis of the ophthalmic data of the participants in dataset 1

## Methods

### Ophthalmic data

Visual field exams were conducted using the Humphrey field analyzer with the 24-2 grid and SITA-Fast strategy (all hemianopic and eight control participants), or the frequency doubling technology (Carl Zeiss Meditec) in C20-1 mode (four control participants). Control exams were used to screen for visual field defects; inclusion required a normal glaucoma hemifield test Humphrey field analyzer or no significant abnormal locations (frequency doubling technology). Structural imaging of the macula and optic nerve head was performed for all participants using optical coherence tomography (Canon HS100 SD-OCT, Tokyo, Japan). Supplementary Figure 1 shows readouts of the Humphrey field analyzer and optical coherent tomography imaging for all hemianopic participants.

### Calculating mean thicknesses of blind and functioning hemiretinas

Using the glaucoma macular imaging protocol, we measured ganglion cell-inner plexiform layer and macular retinal nerve fibre layer thickness in eight retinal segments within a circular region (diameter = 10 mm) centered on the fovea. For the hemianopic participants, this allowed differentiation between 'blind' and 'functioning' hemiretinas, corresponding to the projections from the blind and intact visual fields, respectively (Supplementary Figure 2A). Among the control participants, we adjusted three cases to match the ratio of left- and right-sided hemianopia (8:2) observed in the hemianopic participants by interchanging thickness measures of the left and right retinal segments. For the ganglion cell-inner plexiform layer, we calculated the mean thickness of the blind and functioning hemiretinas in the hemianopic participants by averaging the respective segments. Control data were derived from the corresponding left and right hemiretinal segments of the control participants. For the macular retinal nerve fibre layer, the mean thickness of the blind hemiretina in the hemianopic participants was calculated similarly. For the control participants, the mean thickness of the left hemiretinal segments was used. The mean thickness of the functioning hemiretina in the hemianopic participants' ipsi- and contralesional eyes was calculated separately, with control data from the right hemiretinal segments of the right and left eyes of the control participants.

**
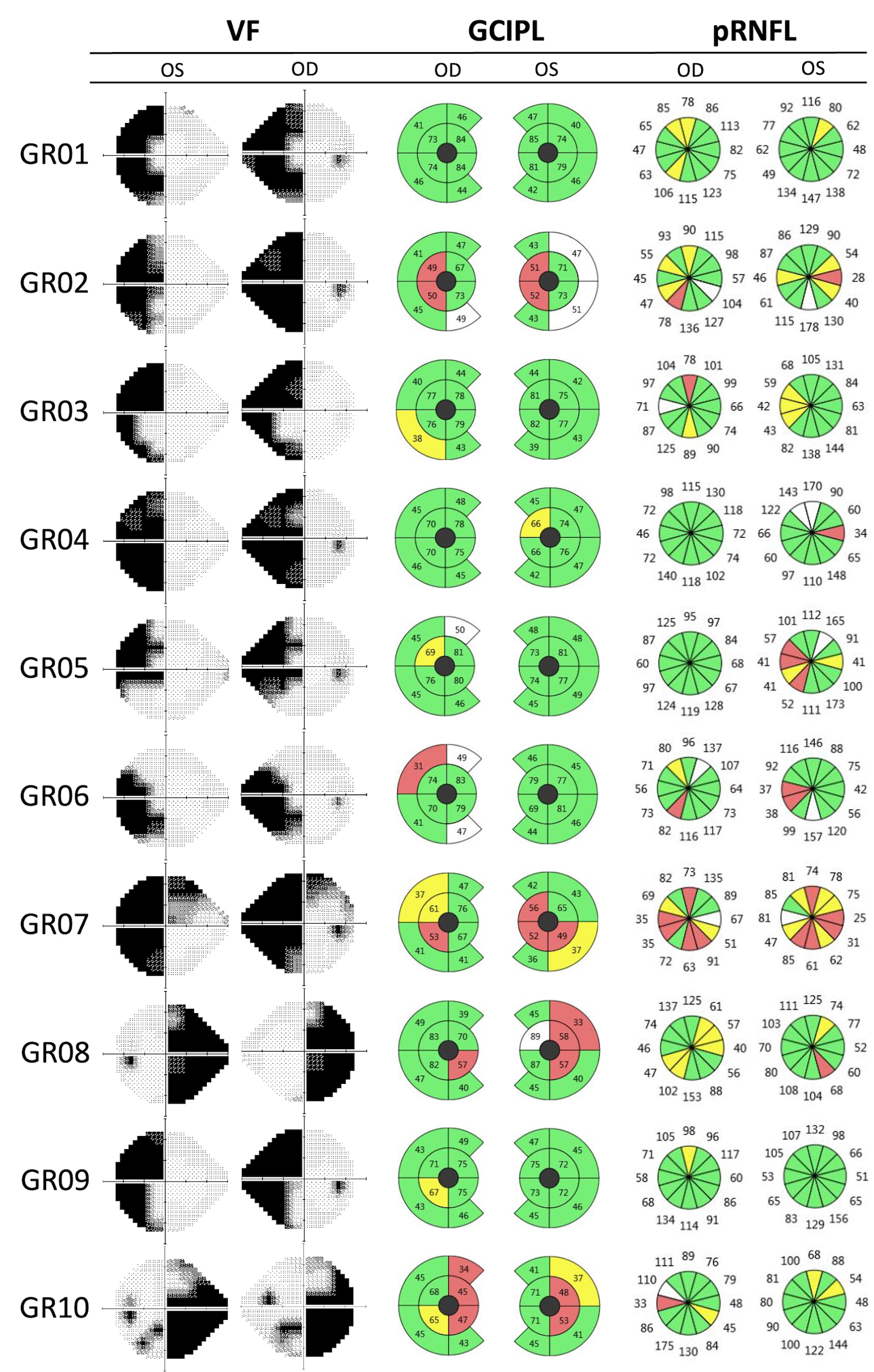
**

**Supplementary Figure 1. Visual field and retinal thickness measures for each hemianopic participant of dataset 1 (GRx, n = 10) illustrating their visual defects and retinal thinning patterns.** Visual field data of the control participants are not shown as they only served a screening purpose. Visual Field (VF) column: Humphrey field analyser test result for the left (OS) and right (OD) eye demonstrating hemianopia. Dark pixels correspond to measures of low contrast sensitivity, indicating a VF defect. Two hemianopic participants, GR01 and GR06, showed macular sparing (i.e., a normal contrast sensitivity within the 10 degrees of fixation). Ganglion cell-inner plexiform layer (GCIPL) and peripapillary retinal nerve fibre layer (pRNFL) columns: optical coherence tomography test results for the right and left eye, demonstrating ganglion cell-inner plexiform layer and peripapillary retinal nerve fibre layer thinning. The colour-coded maps depict how the thickness measurements relate to normal (green or white: within normal limits (p > 5%); yellow: borderline (p < 5%); red: outside normal limits (p < 1%). Visualisations are copies of the Humphrey field analyser test and optical coherence tomography printouts, and use the manufacturer’s built-in statistical algorithms and colour scheme.

Using the glaucoma optic nerve head imaging protocol, we measured peripapillary retinal nerve fibre layer thickness in 12 sectors of a circular region (diameter = 3.45 mm) around the optic nerve head (Supplementary Figure 2B). Mean thickness was calculated for the hemianopic participants' ipsi- and contralesional eyes by averaging the 12 sectors, with control participants’ data based on the same sectors in the right and left eyes. We also analyzed thicknesses for individual optic nerve head sectors in further analyses.


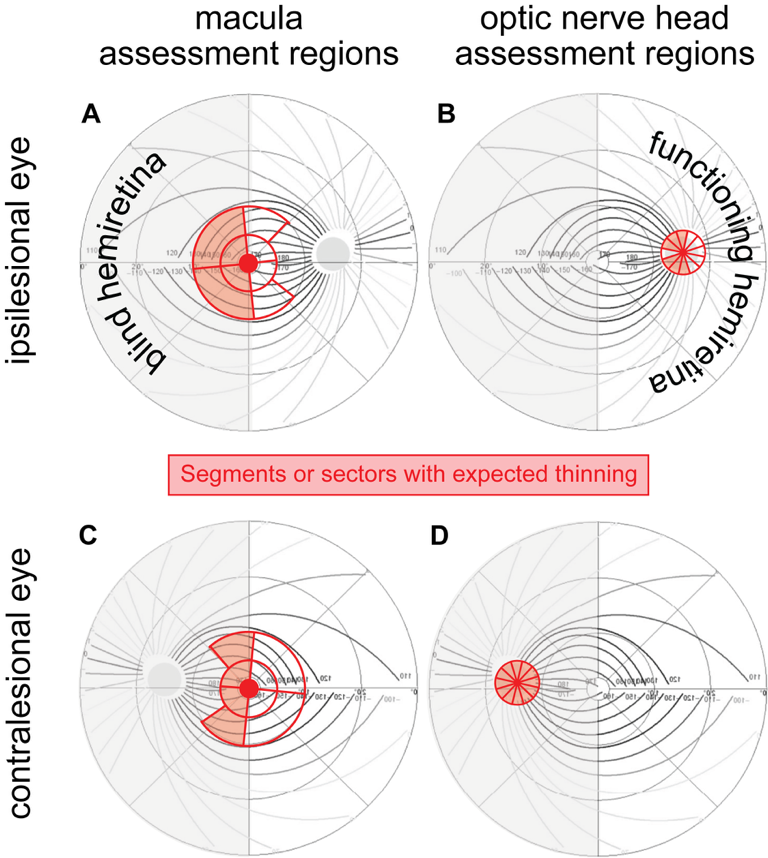
**Supplementary Figure 2. Optical coherence tomography assessment regions.** Assessment regions at the macula (A,C) and at the optic nerve head (B,D), presented on top of a schematic retina representation of the ipsilesional (top row) and contralesional (bottom row) eye. The grey translucent overlays outline the ‘blind’ hemiretinas corresponding to a left-sided hemianopia and right post-geniculate lesion. The grey arcs depict the trajectories of the retinal nerve fibre bundles that come from various retinal locations and assemble at the optic nerve head (modified from Jansonius *et al*.).^1^ The segments (at the macula) and sectors (at the optic nerve head) for which thinning is expected following a right post-geniculate lesion are translucent red. T = temporal side, N = nasal side.

### Retinal thickness analysis

We performed layer-specific comparisons of retinal thickness between the hemiretinas (or eyes) of the hemianopic participants and the corresponding hemiretinas (or eyes) of the control participants. For clarity, we refer to the control participants' matching hemiretina (or eye) as the "control hemiretina (or eye)."

For the ganglion cell-inner plexiform layer, we conducted two separate one-way ANOVAs to compare the thickness of the hemianopic participants' functioning and blind hemiretinas to their control hemiretinas. Additionally, paired t-tests were used to compare the functioning and blind hemiretinas of the hemianopic participants and the two control hemiretinas.

For the macular retinal nerve fibre layer, we conducted a one-way ANOVA to compare the thickness of the blind hemiretina of the hemianopic participants with their control retina. A mixed ANOVA was used to compare the functioning hemiretina of the hemianopic participants to its control retina, for the ipsilesional and contralesional eyes separately. Paired t-tests were also conducted to compare the thicknesses between the two control hemiretinas.

For the peripapillary retinal nerve fibre layer, we conducted two separate one-way repeated-measures ANOVAs to compare 1) the overall thickness and 2) the thickness per sector between the ipsilesional and contralesional eyes of the hemianopic participants and their control eyes. Additionally, paired t-tests were used to compare the thicknesses between the ipsi- and contralesional eyes of the hemianopic participants and the two control eyes.

For all the tests mentioned above, age and gender were included as covariates and statistical significance was set at p < 0.05.

For the hemianopic participants, we performed Pearson linear partial correlations to examine the relationship between 1) the time since injury (in months) and the difference in ganglion cell-inner plexiform layer thickness between the functioning and blind hemiretinas; 2) the time since injury and the average peripapillary retinal nerve fibre layer thickness across both the ipsilesional and contralesional eyes; and 3) ganglion cell-inner plexiform layer thickness of the functioning hemiretina and ipsilesional white matter degeneration (see main manuscript). For the latter two, age (in years) was included as a covariate since retinal thickness naturally declines with age.^1^ The threshold for statistical significance was set at p < 0.05.

## Results

### Macular thinning

Ganglion cell-inner plexiform layer thickness (Supplementary Figure 3, top panel) was significantly reduced in the blind hemiretinas of the hemianopic participants compared to their functioning hemiretinas (mean reduction = 6.8 μm, t(9) = 3.88, p = 0.004, paired t-test) and to the control hemiretinas (mean reduction = 7.0 μm, F(1,18) = 8.24, p = 0.010, Bonferroni corrected, ANOVA). No significant ganglion cell-inner plexiform layer thickness difference was observed between the two control hemiretinas.

Macular retinal nerve fibre layer thickness (Supplementary Figure 3, middle panel) was significantly reduced in the blind hemiretinas of the hemianopic participants compared to the control hemiretinas (mean reduction = 4.8 μm, F(1,18) = 11.52, p = 0.003, Bonferroni corrected, ANOVA). Furthermore, macular retinal nerve fibre layer thickness in the functioning hemiretina of the hemianopic participants was reduced compared to the control hemiretinas for their ipsilesional eye (mean reduction = 13.7 μm, F(1,20) = 53.0, p < 0.001, Greenhouse-Geisser corrected, mixed ANOVA), but not their contralesional eye.

**
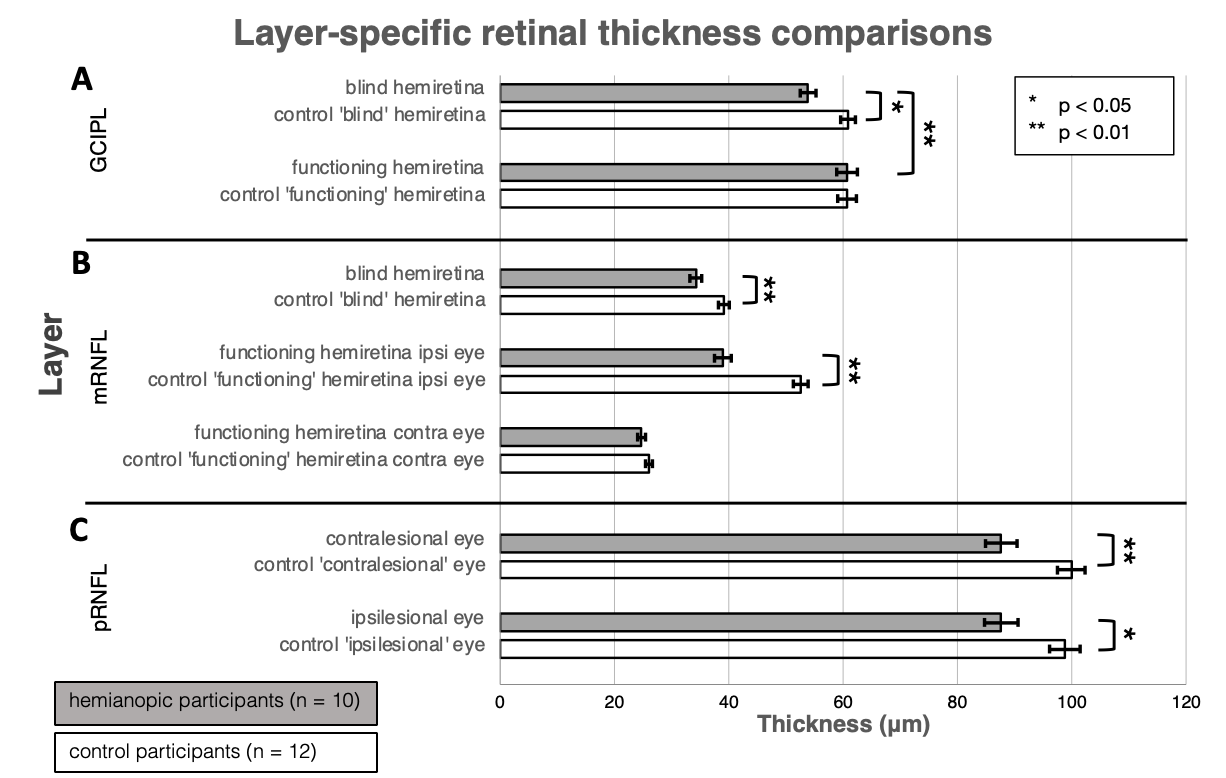
** **Supplementary Figure 3. Layer-specific comparisons of the retinal thicknesses of the hemiretinas and eyes of the hemianopic (n = 10) and control (n = 12) participants.** (**A**) Ganglion cell-inner plexiform layer (GCIPL) thickness difference between the hemianopic participants’ blind hemiretinas and their functioning hemiretinas (mean reduction = 6.8 μm, t(9) = 3.88, p = 0.004, paired t-test) and the control hemiretinas (mean reduction = 7.0 μm, F(1,18) = 8.24, p = 0.010, Bonferroni corrected, ANOVA). (**B**) Macular retinal nerve fibre layer (mRNFL) thickness difference between hemianopic participants’ blind hemiretina and its corresponding control hemiretina (mean reduction = 4.8 μm, F(1,18) = 11.52, p = 0.003, Bonferroni corrected, ANOVA), as well as between their ipsilesional functioning hemiretina and its control hemiretina (F(1,18) = 53.0, p < 0.001, Greenhouse-Geisser corrected, mixed ANOVA). (**C**) Peripapillary retinal nerve fibre layer (pRNFL) thickness differences between the ipsi- and contralesional eyes, and two control eyes (respectively, F(1,18) = 7.54, p = 0.013 and F(1,18) = 11.65, p = 0.003, Bonferroni corrected, ANOVA). Error bars represent the standard error of the means. Statistically significant differences of interest are marked with an asterisk. The gray bars display the thickness averages of the hemianopic participants, the white bars display the thickness averages of the control participants.

### Optic Nerve Head thinning

Peripapillary retinal nerve fibre layer thickness (Supplementary Figure 3, bottom panel) was reduced in both the ipsilesional and contralesional eyes of the hemianopic participants compared to the control eyes, with mean reductions of 11.1 μm and 12.3 μm, respectively (F(1,18) = 7.54, p = 0.013 and F(1,18) = 11.65, p = 0.003, Bonferroni corrected, ANOVA).

For the ipsilesional eye (Supplementary Figure 4, left panel), we observed a significant interaction between participant group and the twelve optic nerve head sectors (F(11) = 5.64, p < 0.01, Greenhouse-Geisser corrected, rmANOVA). Post-hoc analyses showed reduced thickness in the hemianopic compared to control participants in the superior-temporal, superior, inferior-temporal, and temporal-inferior sectors (p < 0.05). No interaction effect was observed for the contralesional eye (F(11) = 0.99, p = 0.42, Greenhouse-Geisser corrected, rmANOVA) (Supplementary Figure 4, right panel).

**
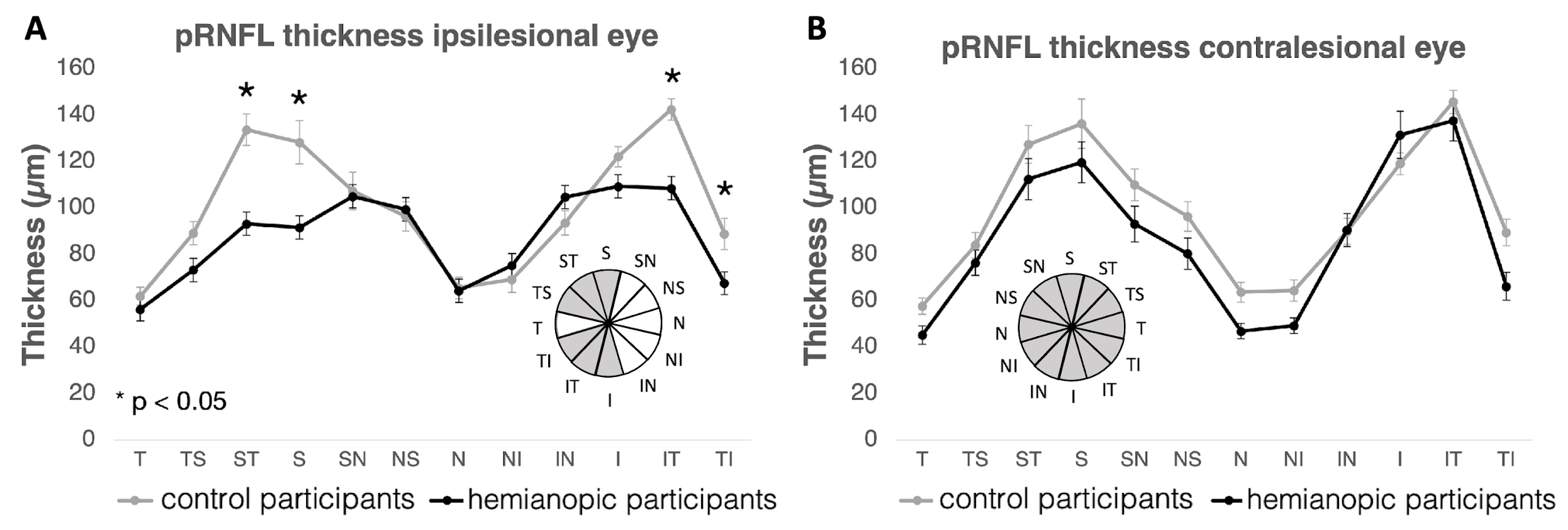
** **Supplementary Figure 4. Sector-specific comparisons of retinal thickness between the hemianopic (n = 10) and control (n = 12) participants of dataset 1.** The two panels display peripapillary retinal nerve fibre layer (pRNFL) thickness (in μm) across optic nerve head sectors for the ipsilesional (**A**) and contralesional (**B**) eyes. (**A**) In the ipsilesional eye, an interaction effect between group (hemianopic vs. control participants) and optic nerve head sectors was observed (F(11) = 5.64, p < 0.01, Greenhouse-Geisser corrected, rmANOVA), with reduced peripapillary retinal nerve fibre layer thickness in the hemianopic participants, specifically in the superior-temporal (ST; mean difference = 33.6 μm, p = 0.002), superior (S; mean difference = 44.5 μm, p = 0.004), inferior-temporal (IT; mean difference = 36.6 μm; p < 0.001), and temporal-inferior (TI; mean difference = 21.5 μm; p = 0.012) sectors compared to the control participants. (**B**) No interaction effect was found in the contralesional eye (F(11) = 0.99, p = 0.42, Greenhouse-Geisser corrected, rmANOVA). Statistically significant differences are indicated with an asterisk. Sectors for which thinning was expected are displayed in grey.

### No relation between time-since-injury and retinal thicknesses

We found no significant correlation between time-since-injury and the ganglion cell-inner plexiform layer thickness difference between the functioning and blind hemiretina (r = -0.15, p = 0.69). Similarly, no significant correlation was observed between time-since-injury and the average peripapillary retinal nerve fibre layer thickness across both the ipsi- and contralateral eyes (r = 0.39, p = 0.30).

**Near-significant associations between ipsilesional white matter changes and retinal thickness**

We observed no statistically significant correlations between ganglion cell–inner plexiform layer (GCIPL) thickness in the functioning hemiretina and reductions in fibre density in the ipsilesional optic tract (r = 0.61, p = 0.08) or the ipsilesional optic radiation (r = 0.60, p = 0.09). However, these near-significant trends suggest a potential relationship that may not have reached significance due to limited statistical power.

### Layer-specific and hemianopic retinal thinning

In the hemianopic participants, we observed thinning of the ganglion cell-inner plexiform layer and macular retinal nerve fibre layer in the blind hemiretinas of both eyes. Notably, additional macular retinal nerve fibre layer thinning was detected in the functioning hemiretina of the ipsilesional eye. This additional thinning likely results from retinal nerve fibre bundles originating from the blind temporal hemiretina that converge at the optic nerve head, as illustrated by the gray arcs in Supplementary Figure 2A.

In addition, we observed peripapillary retinal nerve fibre layer thinning at the optic nerve head in both eyes. In the ipsilesional eye, this thinning was sector-specific with significant reductions in the superior-temporal, superior, inferior-temporal and temporal inferior sectors. These sectors correspond to the fibre bundles originating from the blind hemiretina (see also Supplementary Figure 2B). We found no thinning in sectors containing fibres originating from the functioning hemiretina. In the contralesional eye, we observed overall retinal nerve fibre layer thinning at the optic nerve head. This can be explained by the presence of fibre bundles originating from the blind hemiretina in all sectors, albeit to varying degrees. Overall, this layer-specific retinal thinning demonstrates a topographic pattern that mirrors the hemianopic participants’ visual field defects and supports findings from previous studies.^2-15^

# Supplementary Methods

## Lesion definition

Lesion size and location were manually assessed for each participant of dataset 2 (as described in Willis et al, 2025). Two-four trained researchers manually delineated the lesion on each participant's MRI scan in native space. The lesion was defined as areas of damaged tissue, including damaged white matter. Individual lesion masks were then combined, and a threshold of 1.9 was applied to retain only those voxels identified by at least two researchers, producing a final consensus mask.

## Visual field sensitivity (Total Deviation)

The total deviation in visual field sensitivity represents the difference in sensitivity at each test location compared to age-matched normative values. It is expressed in decibels and is derived from the Humphrey field analyzer. Negative values indicate reduced sensitivity relative to the normal reference. In this study, we calculated the average total deviation within the unaffected hemifield of both eyes. This measure of functional vision allowed us to examine its relationship with the mean reduction in fibre density in the contralesional optic radiation.

## Intracranial volume correction

The fibre-bundle cross-section comparisons were repeated while including intracranial volume (ICV) as a covariate to account for individual differences in brain size. Because ICV and sex are known to be strongly related in the general population, with males typically having larger ICV than females (Ritchie et al., 2018), we examined this relationship in our datasets.

In dataset 2, ICV and sex were correlated (r = 0.36, p = 0.02), whereas in dataset 1, the correlation was not significant (r = 0.33, p = 0.12). To avoid multicollinearity and ensure a consistent approach across analyses, we regressed sex out of ICV in both datasets and used the residuals as covariates in the fibre-bundle cross-section comparisons.

Residuals were computed using a simple linear regression:

*ICV_resid_ = ICV_obs_ – (β_0_ + β_1_ * sex)*

where sex was coded as 0 for female and 1 for male.

Before inclusion in the model, ICV residuals were standardized to z-scores. This step was necessary because the covariates differed substantially in numerical scale: ICV residuals were on the order of hundreds of thousands, age was in tens, and sex was binary (0/1). Such differences in magnitude can inflate the condition number of the design matrix, causing numerical instability during parameter estimation. The *fixelcfestats* command of MRtrix3 interpreted this as near rank deficiency and issued warnings, even though the matrix is not truly rank-deficient and regressors are not collinear. Standardizing covariates resolved this issue by putting ICV residuals on a scale comparable to age and sex, reducing the condition number and improving numerical stability without altering the interpretation of regression coefficients.

# References

1. Jansonius, N. M., Schiefer, J., Nevalainen, J., Paetzold, J. & Schiefer, U. A mathematical model for describing the retinal nerve fiber bundle trajectories in the human eye: average course, variability, and influence of refraction, optic disc size and optic disc position. Exp. Eye Res. 105, 70–78 (2012).
2. Fahrentold, B.K., Cavanaugh, M.R., Tamhankar, M., Lam, B.L., Feldon, S.E., Johson, B.A., & Huxlin, K.R. Training in Cortically Blinded Fields Appears to Confer Patient-Specific Benefit Against Retinal Thinning. Invest. Ophthalmol. Vis. Sci. 64, 4 (2024)
3. Anjos, R. et al. Macular Ganglion Cell Layer and Peripapillary Retinal Nerve Fibre Layer Thickness in Patients with Unilateral Posterior Cerebral Artery Ischaemic Lesion: An Optical Coherence Tomography Study. Neuroophthalmology 40, 8–15 (2016).
4. Goto, K. et al. Sectoral analysis of the retinal nerve fiber layer thinning and its association with visual field loss in homonymous hemianopia caused by post-geniculate lesions using spectral-domain optical coherence tomography. Graefes Arch. Clin. Exp. Ophthalmol. 254, 745–756 (2016).
5. Herro, A. M. & Lam, B. L. Retrograde degeneration of retinal ganglion cells in homonymous hemianopsia. Clin. Ophthalmol. 9, 1057–1064 (2015).
6. Jaumandreu, L., Sánchez-Gutiérrez, V., Muñoz-Negrete, F. J., de Juan, V. & Rebolleda, G. Reduced Peripapillary and Macular Vessel Density in Unilateral Postgeniculate Lesions With Retrograde Transsynaptic Degeneration. J. Neuroophthalmol. 39, 462–469 (2019).
7. Jindahra, P., Petrie, A. & Plant, G. T. Retrograde trans-synaptic retinal ganglion cell loss identified by optical coherence tomography. Brain 132, 628–634 (2009).
8. Jindahra, P., Petrie, A. & Plant, G. T. The time course of retrograde trans-synaptic degeneration following occipital lobe damage in humans. Brain 135, 534–541 (2012).
9. Keller, J., Sánchez-Dalmau, B. F. & Villoslada, P. Lesions in the posterior optic pathway promote trans-synaptic degeneration of retinal ganglion cells. PLoS One 9, e97444 (2014).
10. Mitchell, J. R., Oliveira, C., Tsiouris, A. J. & Dinkin, M. J. Corresponding Ganglion Cell Atrophy in Patients With Postgeniculate Homonymous Visual Field Loss. J. Neuroophthalmol. 35, 353–359 (2015).
11. Mühlemann, F. et al. Homonymous hemiatrophy of ganglion cell layer from retrochiasmal lesions in the optic pathway. Neurology 94, e323–e329 (2020).
12. Yamashita, T. et al. Retinal Ganglion Cell Atrophy in Homonymous Hemianopia due to Acquired Occipital Lesions Observed Using Cirrus High-Definition-OCT. J. Ophthalmol. 2016, 2394957 (2016).
13. Shin, H.-Y., Park, H.-Y. L., Choi, J.-A. & Park, C. K. Macular ganglion cell-inner plexiform layer thinning in patients with visual field defect that respects the vertical meridian. Graefes Arch. Clin. Exp. Ophthalmol. 252, 1501–1507 (2014).
14. Yamashita, T. et al. Preferential atrophy of the central retinal ganglion cells in homonymous hemianopia due to acquired retrogeniculate lesions demonstrated using swept-source optical coherence tomography. Acta Ophthalmol. 96, e538–e539 (2018).
15. Lee, J.-I. et al. Retinal Changes After Posterior Cerebral Artery Infarctions Display Different Patterns of the Nasal und Temporal Sector in a Case Series. Front. Neurol. 11, 508 (2020).
